# (*E*)-3-(4-Fluoro­phen­yl)-1-[4-(methyl­sulfan­yl)phen­yl]prop-2-en-1-one

**DOI:** 10.1107/S1600536808032807

**Published:** 2008-10-15

**Authors:** N. Anuradha, A. Thiruvalluvar, M. Mahalinga, R. J. Butcher

**Affiliations:** a PG Research Department of Physics, Rajah Serfoji Government College (Autonomous), Thanjavur 613 005, Tamil Nadu, India; b SeQuent Scientific Limited, 120 A&B Industrial Area, Baikampady, New Mangalore 575 011, India; c Department of Chemistry, Howard University, 525 College Street NW, Washington, DC 20059, USA

## Abstract

In the title mol­ecule, C_16_H_13_FOS, the dihedral angle between the two benzene rings is 8.68 (6)°. The H atoms of the central enone group are *trans* and one H atom is involved in a close intra­molecular C—H⋯O contact. The crystal structure is stabilized by weak C—H⋯π inter­actions.

## Related literature

For related crystal structures, see: Moorthi *et al.* (2005 ▶); Sathiya Moorthi, *et al.* (2005 ▶); Thiruvalluvar, Subramanyam, Butcher, Adhikari & Wagle (2007 ▶); Thiruvalluvar, Subramanyam, Butcher, Adhikari & Karabasanagouda (2007 ▶); Thiruvalluvar, Subramanyam, Butcher, Karegoudar & Holla (2008 ▶); Thiruvalluvar, Subramanyam, Butcher, Karabasanagouda & Adhikari (2008 ▶). For biological activities of chalcones, see: Anto *et al.* (1995 ▶); Vaya *et al.* (1997 ▶); Mukherjee *et al.* (2001 ▶); Indyah *et al.* (2000 ▶); Chen *et al.* (1997 ▶); Nielsen *et al.* (1998 ▶); Hsin *et al.* (1998 ▶); Kumar *et al.* (2003 ▶); Prasad *et al.* (2005 ▶).
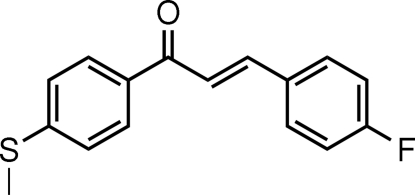


## Experimental

### 

#### Crystal data

C_16_H_13_FOS
*M* *_r_* = 272.33
Monoclinic, 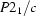

*a* = 29.7846 (9) Å
*b* = 5.7070 (3) Å
*c* = 7.7071 (4) Å
β = 90.781 (3)°
*V* = 1309.94 (10) Å^3^
*Z* = 4
Mo *K*α radiation
μ = 0.25 mm^−1^
*T* = 200 (2) K
0.44 × 0.41 × 0.31 mm

#### Data collection

Oxford Diffraction R Gemini diffractometer
Absorption correction: multi-scan (*CrysAlis RED*; Oxford Diffraction, 2008 ▶) *T*_min_ = 0.945, *T*_max_ = 1.000 (expected range = 0.876–0.926)
11708 measured reflections
4318 independent reflections
2944 reflections with *I* > 2σ(*I*)
*R*_int_ = 0.024

#### Refinement

*R*[*F* ^2^ > 2σ(*F* ^2^)] = 0.042
*wR*(*F* ^2^) = 0.118
*S* = 1.05
4318 reflections
172 parameters
H-atom parameters constrained
Δρ_max_ = 0.31 e Å^−3^
Δρ_min_ = −0.26 e Å^−3^

### 

Data collection: *CrysAlis CCD* (Oxford Diffraction, 2008 ▶); cell refinement: *CrysAlis RED* (Oxford Diffraction, 2008 ▶); data reduction: *CrysAlis RED*; program(s) used to solve structure: *SHELXS97* (Sheldrick, 2008 ▶); program(s) used to refine structure: *SHELXL97* (Sheldrick, 2008 ▶); molecular graphics: *ORTEP-3* (Farrugia, 1997 ▶); software used to prepare material for publication: *PLATON* (Spek, 2003 ▶).

## Supplementary Material

Crystal structure: contains datablocks global, I. DOI: 10.1107/S1600536808032807/lh2707sup1.cif

Structure factors: contains datablocks I. DOI: 10.1107/S1600536808032807/lh2707Isup2.hkl

Additional supplementary materials:  crystallographic information; 3D view; checkCIF report

## Figures and Tables

**Table 1:** Hydrogen-bond geometry (Å, °)

*D*—H⋯*A*	*D*—H	H⋯*A*	*D*⋯*A*	*D*—H⋯*A*
C3—H3⋯O1	0.95	2.42	2.7860 (18)	102
C12—H12⋯*Cg*1^i^	0.95	2.95	3.6803 (16)	134
C15—H15⋯*Cg*1^ii^	0.95	2.87	3.5494 (16)	129
C25—H25⋯*Cg*2^iii^	0.95	2.88	3.5407 (15)	127

Symmetry codes: (i) 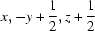; (ii) 
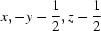
; (iii) 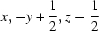. *Cg*1 and *Cg*2 are the centroids of the C11–C16 and C21–C26 rings.

**Fig. 1.**
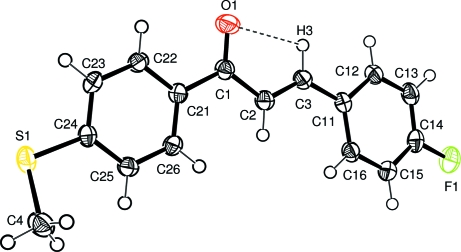
The molecular structure of the title compound, showing the atom-numbering scheme and displacement ellipsoids drawn at the 50% probability level. H atoms are shown as small spheres of arbitrary radius. The dashed line indicates a short intramolecular contact.

## supplementary crystallographic information

### Comment

Chalcones either natural or synthetic are known to exhibit various biological activities. They have been reported to possess antioxidant (Anto *et al.*, 1995; Vaya *et al.*, 1997; Mukherjee *et al.*, 2001; Indyah *et al.*, 2000), antimalarial (Chen *et al.*, 1997), antileishmanial(Nielsen *et al.*, 1998), antiinflammatory(Hsin *et al.*, 1998), antitumor(Kumar *et al.*, 2003) and antibacterial activity(Prasad *et al.*, 2005). The presence of a reactive unsaturated keto function in chalcones is found to be responsible for their antimicrobial activity, which may be altered depending on the type and position of substituent on the aromatic rings.

The title compound (Fig. 1) has been analysed as part of our crystallographic studies on chalcones (Thiruvalluvar, Subramanyam, Butcher, Adhikari & Wagle, 2007; Thiruvalluvar, Subramanyam, Butcher, Adhikari & Karabasanagouda, 2007; Thiruvalluvar, Subramanyam, Butcher, Karegoudar & Holla, 2008; Thiruvalluvar, Subramanyam, Butcher, Karabasanagouda & Adhikari, 2008). The dihedral angle between the two benzene rings is 8.68 (6)°. In similar structures, such as 1-(4-aminophenyl)-3-(3-bromophenyl)-prop-2-en-1-one (Sathiya Moorthi *et al.*, 2005), 1-(4-bromophenyl)-3-(3-hydroxy phenyl)prop-2-en-1-one(Moorthi *et al.*, 2005), the dihedral angles between the two rings are 9.6 (1)° and 10.2 (2)°, respectively. The H atoms of the central enone group are *trans* and one H atom is involved in a close intramolecular C-H···O contact. The crystal structure is stabilized by weak C12—H12···π, C15—H15···π and C25—H25···π interactions (Table 1).

### Experimental

A mixture of 4-acetylthioanisole(16.6 g, 0.1 mol) and 4-fluorobenzaldehyde(12.4 g, 0.1 mol) was stirred in ethanol (30 ml) and then an aqueous solution of KOH (40%, 15 ml) added to it. The mixture was kept overnight at room temperature and then it was poured into crushed ice and acidified with HCl. The solid separated was filtered and crystallized from ethanol. Yield obtained was 85%(23.1 g).

### Refinement

H atoms were positioned geometrically and allowed to ride on their parent atoms, with C—H = 0.95 and 0.98 Å for C*sp*^2^ and methyl C, respectively; *U*_iso_(H) = *xU*_eq_(C), where *x* = 1.5 for methyl H and 1.2 for all other H.

### Crystal data

**Table:**

C_16_H_13_FOS	*F*(000) = 568
*M**_r_* = 272.33	*D*_x_ = 1.381 Mg m^−^^3^
Monoclinic, *P*2_1_/*c*	Melting point: 398(1) K
Hall symbol: -P 2ybc	Mo *K*α radiation, λ = 0.71073 Å
*a* = 29.7846 (9) Å	Cell parameters from 5213 reflections
*b* = 5.7070 (3) Å	θ = 4.7–32.4°
*c* = 7.7071 (4) Å	µ = 0.25 mm^−^^1^
β = 90.781 (3)°	*T* = 200 K
*V* = 1309.94 (10) Å^3^	Chunk, colourless
*Z* = 4	0.44 × 0.41 × 0.31 mm

### Data collection

**Table:**

Oxford Diffraction R Gemini diffractometer	4318 independent reflections
Radiation source: fine-focus sealed tube	2944 reflections with *I* > 2σ(*I*)
graphite	*R*_int_ = 0.024
Detector resolution: 10.5081 pixels mm^-1^	θ_max_ = 32.5°, θ_min_ = 4.7°
φ and ω scans	*h* = −44→43
Absorption correction: multi-scan (CrysAlis RED; Oxford Diffraction, 2008)	*k* = −8→8
*T*_min_ = 0.945, *T*_max_ = 1.000	*l* = −11→11
11708 measured reflections	

### Refinement

**Table:**

Refinement on *F*^2^	Primary atom site location: structure-invariant direct methods
Least-squares matrix: full	Secondary atom site location: difference Fourier map
*R*[*F*^2^ > 2σ(*F*^2^)] = 0.042	Hydrogen site location: inferred from neighbouring sites
*wR*(*F*^2^) = 0.118	H-atom parameters constrained
*S* = 1.05	*w* = 1/[σ^2^(*F*_o_^2^) + (0.0594*P*)^2^ + 0.198*P*] where *P* = (*F*_o_^2^ + 2*F*_c_^2^)/3
4318 reflections	(Δ/σ)_max_ = 0.001
172 parameters	Δρ_max_ = 0.31 e Å^−^^3^
0 restraints	Δρ_min_ = −0.26 e Å^−^^3^

### Special details

**Table:**

Geometry. Bond distances, angles *etc*. have been calculated using the rounded fractional coordinates. All su's are estimated from the variances of the (full) variance-covariance matrix. The cell e.s.d.'s are taken into account in the estimation of distances, angles and torsion angles
Refinement. Refinement of *F*^2^ against ALL reflections. The weighted *R*-factor *wR* and goodness of fit *S* are based on *F*^2^, conventional *R*-factors *R* are based on *F*, with *F* set to zero for negative *F*^2^. The threshold expression of *F*^2^ > 2σ(*F*^2^) is used only for calculating *R*-factors(gt) *etc*. and is not relevant to the choice of reflections for refinement. *R*-factors based on *F*^2^ are statistically about twice as large as those based on *F*, and *R*- factors based on ALL data will be even larger.

### Fractional atomic coordinates and isotropic or equivalent isotropic displacement parameters (Å^2^)

**Table:**

	*x*	*y*	*z*	*U*_iso_*/*U*_eq_	
S1	0.05893 (1)	0.50390 (7)	−0.09311 (5)	0.0340 (1)	
F1	0.46877 (3)	−0.30207 (18)	0.05308 (14)	0.0517 (3)	
O1	0.26356 (3)	0.6477 (2)	0.26067 (15)	0.0456 (4)	
C1	0.25225 (4)	0.4852 (2)	0.16574 (17)	0.0276 (3)	
C2	0.28346 (4)	0.2940 (3)	0.12373 (18)	0.0301 (4)	
C3	0.32640 (4)	0.3052 (3)	0.17803 (17)	0.0280 (4)	
C4	0.04253 (5)	0.2032 (3)	−0.1219 (2)	0.0406 (5)	
C11	0.36238 (4)	0.1383 (2)	0.14776 (16)	0.0261 (3)	
C12	0.40612 (4)	0.1995 (3)	0.19971 (18)	0.0311 (4)	
C13	0.44234 (4)	0.0527 (3)	0.16800 (19)	0.0348 (4)	
C14	0.43389 (4)	−0.1553 (3)	0.08458 (18)	0.0340 (4)	
C15	0.39128 (4)	−0.2253 (3)	0.03214 (18)	0.0320 (4)	
C16	0.35546 (4)	−0.0773 (3)	0.06567 (17)	0.0284 (3)	
C21	0.20523 (4)	0.4821 (2)	0.09295 (15)	0.0236 (3)	
C22	0.17716 (4)	0.6710 (2)	0.12904 (17)	0.0273 (4)	
C23	0.13282 (4)	0.6734 (2)	0.07161 (17)	0.0283 (4)	
C24	0.11552 (4)	0.4850 (2)	−0.02440 (15)	0.0242 (3)	
C25	0.14336 (4)	0.2957 (3)	−0.06185 (17)	0.0284 (4)	
C26	0.18779 (4)	0.2943 (2)	−0.00286 (17)	0.0277 (3)	
H2	0.27322	0.16294	0.05831	0.0361*	
H3	0.33432	0.43933	0.24477	0.0336*	
H4A	0.01108	0.19622	−0.16057	0.0609*	
H4B	0.04601	0.11943	−0.01154	0.0609*	
H4C	0.06157	0.12997	−0.20931	0.0609*	
H12	0.41112	0.34423	0.25774	0.0373*	
H13	0.47196	0.09498	0.20296	0.0418*	
H15	0.38671	−0.37078	−0.02529	0.0384*	
H16	0.32590	−0.12312	0.03247	0.0341*	
H22	0.18857	0.79998	0.19391	0.0327*	
H23	0.11413	0.80338	0.09752	0.0339*	
H25	0.13202	0.16744	−0.12769	0.0340*	
H26	0.20647	0.16408	−0.02808	0.0332*	

### Atomic displacement parameters (Å^2^)

**Table:**

	*U*^11^	*U*^22^	*U*^33^	*U*^12^	*U*^13^	*U*^23^
S1	0.0219 (2)	0.0389 (2)	0.0411 (2)	0.0032 (1)	−0.0038 (1)	0.0049 (2)
F1	0.0332 (4)	0.0550 (6)	0.0669 (6)	0.0198 (4)	−0.0025 (4)	−0.0060 (5)
O1	0.0307 (5)	0.0469 (7)	0.0590 (7)	0.0045 (5)	−0.0097 (5)	−0.0244 (6)
C1	0.0230 (5)	0.0306 (7)	0.0292 (6)	0.0010 (5)	0.0002 (4)	−0.0019 (6)
C2	0.0246 (6)	0.0310 (7)	0.0345 (7)	0.0028 (5)	−0.0026 (5)	−0.0051 (6)
C3	0.0265 (6)	0.0297 (7)	0.0278 (6)	0.0008 (5)	−0.0022 (5)	−0.0016 (5)
C4	0.0280 (7)	0.0479 (10)	0.0459 (8)	−0.0041 (6)	−0.0028 (6)	−0.0090 (7)
C11	0.0224 (5)	0.0306 (7)	0.0251 (6)	0.0008 (5)	−0.0025 (4)	0.0031 (5)
C12	0.0275 (6)	0.0317 (7)	0.0340 (7)	−0.0016 (5)	−0.0065 (5)	0.0002 (6)
C13	0.0235 (6)	0.0412 (9)	0.0396 (7)	0.0003 (5)	−0.0061 (5)	0.0039 (7)
C14	0.0258 (6)	0.0382 (8)	0.0378 (7)	0.0091 (5)	−0.0013 (5)	0.0040 (6)
C15	0.0306 (6)	0.0294 (7)	0.0360 (7)	0.0026 (5)	−0.0032 (5)	−0.0003 (6)
C16	0.0241 (5)	0.0302 (7)	0.0309 (6)	−0.0008 (5)	−0.0037 (5)	0.0028 (6)
C21	0.0216 (5)	0.0252 (7)	0.0239 (5)	0.0007 (5)	0.0023 (4)	0.0011 (5)
C22	0.0260 (6)	0.0239 (7)	0.0319 (6)	0.0007 (5)	0.0008 (5)	−0.0023 (5)
C23	0.0276 (6)	0.0239 (7)	0.0333 (7)	0.0062 (5)	0.0021 (5)	0.0000 (5)
C24	0.0211 (5)	0.0285 (7)	0.0230 (5)	0.0018 (5)	0.0012 (4)	0.0038 (5)
C25	0.0261 (6)	0.0286 (7)	0.0303 (6)	0.0005 (5)	−0.0016 (5)	−0.0060 (5)
C26	0.0235 (5)	0.0276 (7)	0.0319 (6)	0.0046 (5)	0.0011 (5)	−0.0045 (5)

### Geometric parameters (Å, °)

**Table:**

S1—C4	1.7972 (17)	C23—C24	1.3995 (17)
S1—C24	1.7631 (12)	C24—C25	1.3945 (19)
F1—C14	1.3589 (17)	C25—C26	1.3934 (17)
O1—C1	1.2256 (17)	C2—H2	0.9500
C1—C2	1.4724 (19)	C3—H3	0.9500
C1—C21	1.5016 (17)	C4—H4A	0.9800
C2—C3	1.3418 (17)	C4—H4B	0.9800
C3—C11	1.4550 (19)	C4—H4C	0.9800
C11—C12	1.4019 (17)	C12—H12	0.9500
C11—C16	1.398 (2)	C13—H13	0.9500
C12—C13	1.390 (2)	C15—H15	0.9500
C13—C14	1.372 (2)	C16—H16	0.9500
C14—C15	1.3855 (18)	C22—H22	0.9500
C15—C16	1.388 (2)	C23—H23	0.9500
C21—C22	1.3946 (16)	C25—H25	0.9500
C21—C26	1.3976 (17)	C26—H26	0.9500
C22—C23	1.3872 (17)		
			
S1···H4A^i^	3.0100	H2···C16	2.8100
S1···H4C^ii^	3.0600	H2···C26	2.6900
S1···H23^iii^	3.1200	H2···H16	2.2700
F1···F1^iv^	3.0456 (14)	H2···H26	2.0900
F1···H13^v^	2.6300	H2···O1^viii^	2.9100
O1···H3	2.4200	H3···O1	2.4200
O1···H16^vi^	2.8900	H3···C15^vi^	3.0500
O1···H22	2.4400	H3···H12	2.3500
O1···H2^ii^	2.9100	H3···C16^ii^	2.6600
O1···H16^ii^	2.7800	H3···H16^ii^	2.4700
C1···C26^ii^	3.5900 (18)	H4A···S1^x^	3.0100
C3···C15^vi^	3.498 (2)	H4B···C25	3.1000
C3···C16^ii^	3.466 (2)	H4C···C25	2.8400
C12···C15^vi^	3.553 (2)	H4C···H25	2.1900
C15···C12^vii^	3.553 (2)	H4C···S1^viii^	3.0600
C15···C3^vii^	3.498 (2)	H4C···C24^viii^	3.0000
C16···C3^viii^	3.466 (2)	H12···C15^vi^	3.0600
C21···C26^ii^	3.5364 (17)	H12···H3	2.3500
C26···C21^viii^	3.5364 (17)	H12···C15^ii^	3.1000
C26···C1^viii^	3.5900 (18)	H13···F1^xi^	2.6300
C1···H26^ii^	2.8700	H15···C3^vii^	3.0300
C2···H26	2.6700	H15···C12^vii^	3.0500
C2···H16	2.7900	H15···C11^xii^	3.0300
C3···H15^vi^	3.0300	H15···C12^xii^	2.8900
C4···H25	2.6700	H15···C13^xii^	3.0900
C11···H15^ix^	3.0300	H16···O1^vii^	2.8900
C12···H15^ix^	2.8900	H16···C2	2.7900
C12···H15^vi^	3.0500	H16···H2	2.2700
C13···H15^ix^	3.0900	H16···O1^viii^	2.7800
C15···H12^viii^	3.1000	H16···H3^viii^	2.4700
C15···H3^vii^	3.0500	H22···O1	2.4400
C15···H12^vii^	3.0600	H23···S1^xiii^	3.1200
C16···H2	2.8100	H25···C4	2.6700
C16···H3^viii^	2.6600	H25···H4C	2.1900
C21···H26^ii^	3.0400	H25···C22^viii^	3.0200
C22···H25^ii^	3.0200	H25···C23^viii^	3.0300
C23···H25^ii^	3.0300	H26···C2	2.6700
C24···H4C^ii^	3.0000	H26···H2	2.0900
C25···H4B	3.1000	H26···C1^viii^	2.8700
C25···H4C	2.8400	H26···C21^viii^	3.0400
C26···H2	2.6900		
			
C4—S1—C24	103.64 (6)	C1—C2—H2	120.00
O1—C1—C2	121.52 (11)	C3—C2—H2	120.00
O1—C1—C21	118.64 (11)	C2—C3—H3	116.00
C2—C1—C21	119.84 (11)	C11—C3—H3	116.00
C1—C2—C3	119.87 (14)	S1—C4—H4A	109.00
C2—C3—C11	128.30 (14)	S1—C4—H4B	109.00
C3—C11—C12	118.34 (12)	S1—C4—H4C	109.00
C3—C11—C16	122.99 (11)	H4A—C4—H4B	109.00
C12—C11—C16	118.65 (12)	H4A—C4—H4C	109.00
C11—C12—C13	121.33 (14)	H4B—C4—H4C	109.00
C12—C13—C14	117.78 (12)	C11—C12—H12	119.00
F1—C14—C13	118.77 (11)	C13—C12—H12	119.00
F1—C14—C15	118.01 (14)	C12—C13—H13	121.00
C13—C14—C15	123.21 (13)	C14—C13—H13	121.00
C14—C15—C16	118.27 (15)	C14—C15—H15	121.00
C11—C16—C15	120.74 (12)	C16—C15—H15	121.00
C1—C21—C22	118.36 (10)	C11—C16—H16	120.00
C1—C21—C26	122.99 (10)	C15—C16—H16	120.00
C22—C21—C26	118.60 (11)	C21—C22—H22	120.00
C21—C22—C23	120.98 (11)	C23—C22—H22	119.00
C22—C23—C24	120.21 (11)	C22—C23—H23	120.00
S1—C24—C23	117.09 (9)	C24—C23—H23	120.00
S1—C24—C25	123.64 (9)	C24—C25—H25	120.00
C23—C24—C25	119.27 (11)	C26—C25—H25	120.00
C24—C25—C26	120.11 (13)	C21—C26—H26	120.00
C21—C26—C25	120.83 (12)	C25—C26—H26	120.00
			
C4—S1—C24—C23	−155.77 (10)	C12—C13—C14—F1	−179.65 (13)
C4—S1—C24—C25	24.79 (13)	C12—C13—C14—C15	−0.4 (2)
O1—C1—C2—C3	−5.5 (2)	F1—C14—C15—C16	179.30 (13)
C21—C1—C2—C3	175.08 (12)	C13—C14—C15—C16	0.1 (2)
O1—C1—C21—C22	3.39 (18)	C14—C15—C16—C11	1.0 (2)
O1—C1—C21—C26	−173.80 (12)	C1—C21—C22—C23	−177.24 (11)
C2—C1—C21—C22	−177.22 (12)	C26—C21—C22—C23	0.08 (18)
C2—C1—C21—C26	5.60 (18)	C1—C21—C26—C25	177.44 (12)
C1—C2—C3—C11	−178.78 (13)	C22—C21—C26—C25	0.26 (19)
C2—C3—C11—C12	172.54 (15)	C21—C22—C23—C24	−0.13 (19)
C2—C3—C11—C16	−5.9 (2)	C22—C23—C24—S1	−179.63 (10)
C3—C11—C12—C13	−177.20 (13)	C22—C23—C24—C25	−0.16 (18)
C16—C11—C12—C13	1.3 (2)	S1—C24—C25—C26	179.91 (10)
C3—C11—C16—C15	176.76 (13)	C23—C24—C25—C26	0.48 (19)
C12—C11—C16—C15	−1.7 (2)	C24—C25—C26—C21	−0.5 (2)
C11—C12—C13—C14	−0.3 (2)		

Symmetry codes: (i) −*x*, *y*+1/2, −*z*−1/2; (ii) *x*, −*y*+1/2, *z*+1/2; (iii) *x*, −*y*+3/2, *z*−1/2; (iv) −*x*+1, −*y*−1, −*z*; (v) −*x*+1, *y*−1/2, −*z*+1/2; (vi) *x*, *y*+1, *z*; (vii) *x*, *y*−1, *z*; (viii) *x*, −*y*+1/2, *z*−1/2; (ix) *x*, −*y*−1/2, *z*+1/2; (x) −*x*, *y*−1/2, −*z*−1/2; (xi) −*x*+1, *y*+1/2, −*z*+1/2; (xii) *x*, −*y*−1/2, *z*−1/2; (xiii) *x*, −*y*+3/2, *z*+1/2.

### Hydrogen-bond geometry (Å, °)

**Table:**

*D*—H···*A*	*D*—H	H···*A*	*D*···*A*	*D*—H···*A*
C3—H3···O1	0.95	2.42	2.7860 (18)	102
C12—H12···Cg1^ii^	0.95	2.95	3.6803 (16)	134
C15—H15···Cg1^xii^	0.95	2.87	3.5494 (16)	129
C25—H25···Cg2^viii^	0.95	2.88	3.5407 (15)	127

Symmetry codes: (ii) *x*, −*y*+1/2, *z*+1/2; (xii) *x*, −*y*−1/2, *z*−1/2; (viii) *x*, −*y*+1/2, *z*−1/2.

## References

[bb1] Anto, R. J., Sukumaran, K., Kuttan, G., Rao, M. N. A., Subbaraju, V. & Kuttan, R. (1995). *Cancer Lett.* **97**, 33–36. 10.1016/0304-3835(95)03945-s 7585475

[bb2] Chen, M., Christensen, S. B., Zhai, L., Rasmussen, M. H., Theander, T. G., Frokjaer, S., Steffensen, B., Davidson, J. & Kharazmi, A. J. (1997). *Infect. Dis.* **176**, 1327–1330. 10.1086/514129 9359735

[bb3] Farrugia, L. J. (1997). *J. Appl. Cryst.* **30**, 565.

[bb4] Hsin, H.-K., Tai, L.-H., Pyang, J.-W., Jey, W.-J. & Chun, L.-N. (1998). *Pharm. Res.* **15**, 39–42.

[bb5] Indyah, S. A., Timmerman, H., Samhoedi, M., Sastrohami, D., Sugiyanto, H. & Van Der Goot, H. (2000). *Eur. J. Med. Chem.* **35**, 449–452. 10.1016/s0223-5234(00)00137-9 10858605

[bb6] Kumar, S. K., Hager, E., Catherine, P., Gurulingappa, H., Davidson, N. E. & Khan, S. R. (2003). *J. Med. Chem.* **46**, 2813–2815. 10.1021/jm030213+ 12825923

[bb7] Moorthi, S. S., Chinnakali, K., Nanjundan, S., Unnithan, C. S., Fun, H.-K. & Yu, X.-L. (2005). *Acta Cryst.* E**61**, o483–o485.

[bb8] Mukherjee, S., Kumar, V., Prasad, A. K., Raj, H. G., Brakhe, M. E., Olsen, C. E., Jain, S. C. & Parmar, V. P. (2001). *Bioorg. Med. Chem.* **9**, 337–339. 10.1016/s0968-0896(00)00249-2 11249126

[bb9] Nielsen, S. F., Christensen, S. B., Cruciani, G., Kharazmi, A. & Liljefors, T. (1998). *J. Med. Chem.* **41**, 4819–4822. 10.1021/jm980410m 9822551

[bb10] Oxford Diffraction (2008). *CrysAlis CCD* and *CrysAlis RED* Oxford Diffraction Ltd, Abingdon, Oxfordshire, England.

[bb11] Prasad, Y. R., Prasoona, L., Rao, A. L., Lakshmi, K., Kumar, P. R. & Rao, B. G. (2005). *Int. J. Chem. Sci.* **3**, 685–689.

[bb12] Sathiya Moorthi, S., Chinnakali, K., Nanjundan, S., Santhi, R. & Fun, H.-K. (2005). *Acta Cryst.* E**61**, o3514–o3516.

[bb13] Sheldrick, G. M. (2008). *Acta Cryst.* A**64**, 112–122. 10.1107/S0108767307043930 18156677

[bb14] Spek, A. L. (2003). *J. Appl. Cryst.* **36**, 7–13.

[bb15] Thiruvalluvar, A., Subramanyam, M., Butcher, R. J., Adhikari, A. V. & Karabasanagouda, T. (2007). *Acta Cryst.* E**63**, o4716. 10.1107/S1600536808017200 PMC2961704 21202897

[bb16] Thiruvalluvar, A., Subramanyam, M., Butcher, R. J., Adhikari, A. V. & Wagle, S. (2007). *Acta Cryst.* E**63**, o4536.

[bb17] Thiruvalluvar, A., Subramanyam, M., Butcher, R. J., Karabasanagouda, T. & Adhikari, A. V. (2008). *Acta Cryst.* E**64**, o1263. 10.1107/S1600536808017200 PMC2961704 21202897

[bb18] Thiruvalluvar, A., Subramanyam, M., Butcher, R. J., Karegoudar, P. & Holla, B. S. (2008). *Acta Cryst.* E**64**, o60. 10.1107/S1600536807062010 PMC2915018 21200937

[bb19] Vaya, R., Belinky, P. A. & Aviram, M. (1997). *Free Radic. Biol. Med.* **23**, 302–305. 10.1016/s0891-5849(97)00089-0 9199893

